# Mother–Child Closeness Trajectories in Families of Children With Intellectual Disabilities From a UK Cohort Study

**DOI:** 10.1111/jir.70122

**Published:** 2026-05-27

**Authors:** Emma L. Taylor, Paul A. Thompson, Samantha Flynn, Kylie M. Gray, Richard P. Hastings

**Affiliations:** ^1^ Intellectual Disabilities Research Institute (IDRIS) University of Birmingham Edgbaston UK; ^2^ Centre for Research in Intellectual and Developmental Disabilities University of Warwick Coventry UK; ^3^ Department of Psychiatry, School of Clinical Sciences at Monash Health Monash University Melbourne Australia

**Keywords:** families, intellectual disabilities, mother–child closeness, trajectories

## Abstract

**Background:**

The closeness of the parent–child relationship may be a crucial factor in the development of all children, including those with intellectual disabilities. Previous research has suggested that there may be subgroup trajectories of mother–child closeness in families of children with intellectual disabilities during childhood. Our aim was to examine whether potential subgroup trajectories of mother–child closeness in families of children with intellectual disabilities exist and what factors influence identified trajectories.

**Methods:**

Data from 353 maternal primary caregivers who took part in three waves of the 1000 Families Study, over the course of 8 years, were analysed using growth mixture modelling. Mother–child closeness was measured using the Child Parent Relationship Scale at all three waves. Time‐varying (maternal psychological distress, child behaviour and emotional problems, child prosocial behaviour) and time‐invariant (child's level of communication skills, autism diagnosis) correlates of class membership and within‐class trajectories were included in the analysis.

**Results:**

The findings suggested a single trajectory of mother–child closeness, with observed heterogeneity accounted for by the covariates. Mother–child closeness on average remained consistent over time. Maternal psychological distress and an autism diagnosis were generally associated with reduced levels of mother–child closeness. The child's level of communication skills and child prosocial behaviour were associated with increased levels of closeness over time. Child behaviour and emotional problems were not associated with mother–child closeness.

**Conclusion:**

Further research using larger longitudinal population datasets is needed to explore whether there may be distinct trajectories of mother–child closeness in families of children with intellectual disabilities. The current findings suggest that supports for mother–child relationships might be targeted in families of children with lower levels of communication or prosocial behaviour skills, an additional autism diagnosis and when mothers are experiencing higher levels of psychological distress.

## 1 Introduction

According to attachment theory, a warm, close parent–child relationship, whereby the parent provides consistent, sensitive and responsive caregiving, can enhance children's long‐term psychological, social and emotional outcomes (Bowlby 1977). In particular, children who form a close parent–child relationship are more likely to feel a greater sense of self‐worth (Bulanda and Majumdar 2009; McAdams et al. 2017), be more trusting of others (Rotenberg et al. 2021) and develop positive peer relationships (Lee 2018; Schneider et al. 2001). In contrast, difficulties in forming a close relationship are associated with an increased risk of child behavioural and emotional problems (Dreidi et al. 2024; Groh et al. 2017; Fearon et al. 2010), which may have wider societal and financial implications (Bachmann et al. 2019). Therefore, considering the role of the parent–child relationship—in particular, parent–child closeness—is crucial in the context of children's development.

In families of children with intellectual disabilities, a close parent–child relationship is similarly recognised as important for children's behavioural and emotional outcomes. Findings from UK population cohort studies have shown that a close parent–child relationship has been associated with fewer child behaviour and emotional problems (Totsika et al. 2014, 2020) and increases in child prosocial behaviour over time (Williams et al. 2025), with the quality of this relationship having a greater influence on children's outcomes than parenting behaviours (Schuiringa et al. 2015; Williams et al. 2025). However, previous cross‐sectional research has suggested that children with intellectual disabilities may have a less close mother–child relationship when compared with other children. For example, Totsika et al. (2014) found that mothers of children with intellectual disabilities living in the UK reported less closeness and increased levels of conflict with their child than mothers of children without intellectual disabilities when the child was 3 years of age. Similarly, Aksoy and Kobya Bulut (2025) reported that, when the child was aged between 6 and 18 years, mothers of children with intellectual disabilities living in Turkey reported a less close relationship with their child compared with mothers of non‐disabled children. However, as data were only collected at one time point, these findings are limited in capturing how mother–child closeness changes over time in families of children with intellectual disabilities. Given the importance of a close relationship for children's later outcomes, understanding how this relationship develops over time may help to inform the implementation of early intervention support during childhood.

Although previous research has examined the developmental pattern of maternal parenting behaviours (e.g., Fenning et al. 2014; Vilaseca et al. 2023) and mother–child conflict (e.g., Marquis et al. 2017) in families of children with intellectual disabilities, relatively few studies have examined how mother–child closeness develops and changes over time. Fielding‐Gebhardt et al. (2020) examined the trajectory of mother–child closeness in 55 families with a child with Fragile X syndrome and found no significant overall rates of change in closeness when measured at three time points. Taylor et al. (2026) similarly found that the trajectory of mother–child closeness remained stable over time when examining three waves of data from 353 families of children with intellectual disabilities. However, both studies reported significant heterogeneity in closeness over time, suggesting that mother–child closeness may vary amongst families and that examining individual‐level change may be more informative than average trajectory growth. Examining whether subgroup trajectories of mother–child closeness exist, and the factors associated with these trajectories, may help to identify families who are at risk of developing a less close mother–child relationship in families of children with intellectual disabilities.

To the best of the authors' knowledge, no research to date has examined for subgroup trajectories of mother–child closeness in families of children with intellectual disabilities. However, distinct trajectories of mother–child closeness/warmth have been explored in families of children without intellectual disabilities. Using growth mixture modelling (GMM), Seiffge‐Krenke et al. (2010) examined changes in mother–child relationship quality (closeness/support and negative affect) over time in a sample of German families during adolescence, using four waves of data. Upon testing up to 4‐class models, Seiffge‐Krenke et al. found three subgroup trajectories for mother–child relationships. Families in the first and largest subgroup reported initial high levels of closeness/support and negative affect with their child, with a slight decline over time. A second subgroup included those who had lower levels of closeness/support with their child at baseline, which declined over time, whilst negative affect increased over time. A small third subgroup was identified for mothers who reported high levels of negative affect and low levels of closeness/support with their child at baseline, both of which decreased over time. Additionally, using group‐based trajectory modelling, Buckley et al. (2024) analysed data from the Longitudinal Study of Australian Children to examine subgroup trajectories of warmth in the mother–son relationship using seven waves of data (between the ages of 4–5 and 16–17 years). Similar to Seiffge‐Krenke et al., three distinct trajectory subgroups of parental warmth were reported (those who display high levels of warmth over time, those with some small decline and those with a steady decline over time), indicating the presence of unobserved heterogeneity amongst families. The findings from these studies therefore highlight the usefulness of examining whether similar distinct subgroup trajectories are present in families of children with intellectual disabilities to identify families who may require additional support targeting mother–child closeness.

Given this gap in the current literature, the present study aimed to examine for subgroup trajectories of mother–child closeness in families of children with intellectual disabilities. Specifically, this study aimed to address the following research questions: (1) Are there different trajectories of mother–child closeness in families of children with intellectual disabilities? and (2) what factors are associated with any identified trajectories of mother–child closeness in families of children with intellectual disabilities? We hypothesised that there would be variations in levels of mother–child closeness amongst families of children with intellectual disabilities; thus, there will be subgroup trajectories of mother–child closeness present. Furthermore, we hypothesised that maternal psychological distress, child behaviour and emotional problems, and a diagnosis of autism would be associated with reduced levels of mother–child closeness over time, whereas child prosocial behaviour and the child's level of communication skills would be positively associated with mother–child closeness over time.

## 2 Methods

### 2.1 Study Design

This study drew upon three waves of data from the 1000 Families Study: a UK longitudinal cohort study (Hastings et al. 2020). At Wave 1, the 1000 Families Study recruited families living in the UK who have a child with an intellectual disability aged between 4 years and 15 years and 11 months (Hastings et al. 2020). Informed consent obtained for the 1000 Families Study also covers secondary research, such as this study. Further information on the recruitment and consent process can be found in Hastings et al. (2020).

### 2.2 Participants

To address the aims of this study, we were interested in analysing data provided by maternal primary caregivers at all three waves. In the 1000 Families Study, data were provided by 1184 families at Wave 1 (2015–2017), 650 families at Wave 2 (2018–2021) and 577 families at Wave 3 (2021–2023), with 509 families taking part at all three waves. Following Taylor et al. (2026), we excluded *n* = 20 families where the main respondent did not identify as a maternal caregiver at any of the three waves. In addition, we excluded *n* = 136 families whose child with intellectual disabilities was aged 16 or over at Wave 3, as children's behaviour was not measured in this survey. This resulted in a final sample of 353 participants. Table 1 displays demographic information for these families.

**TABLE 1:** Maternal caregiver and child demographic information at Wave 1.

Variable	*N* (%)
Caregiver relationship to the child	
Biological mother	330 (93.5%)
Adoptive mother	14 (4%)
Grandmother	8 (2.3%)
Foster mother	1 (0.3%)
Caregiver ethnicity	
White British	310 (87.8%)
White (Irish/travelling community/other)	21 (6%)
Asian/Asian British (Indian/Pakistani/Bangladeshi/Chinese/other)	9 (2.6%)
Multiple/mixed ethnic groups	7 (2%)
Black (African/Caribbean/Black British/other)	3 (0.9%)
Missing data	3 (0.9%)
Caregiver marital status	
Married	224 (63.5%)
Living with partner	57 (16.2%)
Not married or not currently living with partner	56 (15.9%)
Missing data	16 (4.5%)
Child sex (Wave 1)	
Male	244 (69.1%)
Female	107 (30.3%)
Missing data	2 (0.6%)
Additional diagnoses	
Autism (parent reported)	236 (67%)
Child age (years)	M (SD)
Wave 1	7.33 (1.91)
Wave 2	10.31 (1.98)
Wave 3	12.94 (1.92)

### 2.3 Measures

#### 2.3.1 Mother–Child Closeness

Mother–child closeness was measured at all three waves using the Child Parent Relationship Scale–Short Form (CPRS‐SF; Pianta 1995). The closeness subscale consists of seven items that are scored using a 5‐point Likert‐type scale, ranging from 1 *= Definitely does not apply* to 5 = *Definitely applies*. A total perceived closeness score was generated by summing the scores for all seven items; a higher total score indicates increased perceived mother–child closeness.

In previous research with families of children with intellectual disabilities, the CPRS‐SF has shown acceptable internal consistency (Totsika et al. 2020; Zabidi et al. 2023). In the current study, McDonald's omega for the closeness subscale demonstrated acceptable internal consistency at Wave 1 (*ω* = 0.78).

As pre‐registered (https://osf.io/qf9ws/), we accounted for time‐varying and time‐invariant child and parental factors that may be important for the development of mother–child closeness over time. Previous research has shown that reduced levels of closeness have been associated with increased levels of maternal psychological distress (Taylor et al. 2026; Totsika et al. 2020; Zabidi et al. 2023), a diagnosis of autism (Taylor et al. 2026; Teague et al. 2018) and child behaviour and emotional problems (Fielding‐Gebhardt et al. 2020), whilst both the child's level of communication skills and child prosocial behaviour have been positively associated with mother–child closeness over time (Taylor et al. 2026).

#### 2.3.2 Time‐Varying Covariates

##### 2.3.2.1 Maternal Psychological Distress

Maternal psychological distress was measured at all three waves using the Kessler 6 (K6; Kessler et al. 2002). Participants rated how often they experienced certain symptoms in the last 30 days using a 5‐point Likert‐type scale (0 = *None of the time* to 4 = *All of the time*). A total score was calculated by summing the scores from all six items, with higher scores indicative of greater levels of maternal psychological distress. In the current study, the K6 showed good internal consistency at Wave 1 (*ω* = 0.88).

##### 2.3.2.2 Child Behavioural and Emotional Problems and Prosocial Behaviour

Child behavioural and emotional problems and prosocial behaviour were measured using the Strengths and Difficulties Questionnaire (SDQ; Goodman 1997). At all three waves, participants were asked to respond to 25 items about the child's behaviour in the last 6 months using a 3‐point scale (0 = *Not true* to 2 = *Certainly true*). A total difficulties score was calculated by summing the scores for 20 SDQ problem items relating to four problem domains: hyperactivity (five items), conduct problems (five items), emotional problems (five items) and peer relationship problems (five items); a total prosocial behaviour score was established by summing the scores on five items (Goodman 1997). Higher total difficulties scores are indicative of greater child behavioural and emotional problems, and higher prosocial scores indicate more child prosocial behaviour. The SDQ is reported to be a valid and reliable measure for identifying behaviour and emotional problems in children with intellectual disabilities (Murray et al. 2021). At Wave 1, the SDQ total difficulties score (*ω* = 0.82) and the prosocial behaviour subscale (*ω* = 0.82) displayed good internal consistency.

#### 2.3.3 Time‐Invariant Covariates

##### 2.3.3.1 Child Diagnosis of Autism

Participants reported if their child with intellectual disabilities had also received an autism diagnosis. Their child was included in the analysis as also being autistic if an autism diagnosis was reported at any of the three waves. In total, 236 children (67%) were included in the analysis as having a diagnosis of autism.

##### 2.3.3.2 Child Communication Skills

The child's level of communication skills was measured using data collected at Waves 2 and 3 only. At both waves, participants were asked to respond to two communication items from the GO4KIDDS Brief Adaptive Scale: ‘How much does your children understanding spoken language?’ and ‘How much does your child use spoken language to communicate?’ (Perry et al. 2015). Participants were also asked to respond to a third item measuring the child's use of alternative methods of communication (‘How much does your child use alternative methods of communication (e.g., signing, Makaton, symbol systems, PECS) to communicate?’). Participants were asked to respond to all items using a 5‐point scale, with 1 representative of very little communication skills and 5 indicative of greater communication skills (e.g., 1 = *Able to understand very little spoken language* and 5 = *Able to understand complex language about a wide range of topics*). Similar to Taylor et al. (2026), to accurately capture the child's overall level of communication skills, a total communication skills score was created by summing the score for the child's receptive communication skills and the higher of the scores for either the child's use of alternative methods of communication skills or child's spoken communication skills. Data from the wave with the highest total communication score (i.e., Wave 2 or Wave 3) were used in the analysis to reflect the child's highest level of communication and thus provide an estimate of their highest level of communication skills, which would be lost if an average across time points was used.

### 2.4 Statistical Analysis

GMM was carried out to (i) explore whether there are different trajectories of mother–child closeness in families of children with intellectual disabilities and (ii) examine what factors are associated with any identified trajectories. All analyses were conducted using the statistical analysis programme, R (Version 4.2.3), using the *lcmm* package (Proust‐Lima et al. 2017).

**FIGURE 1:**
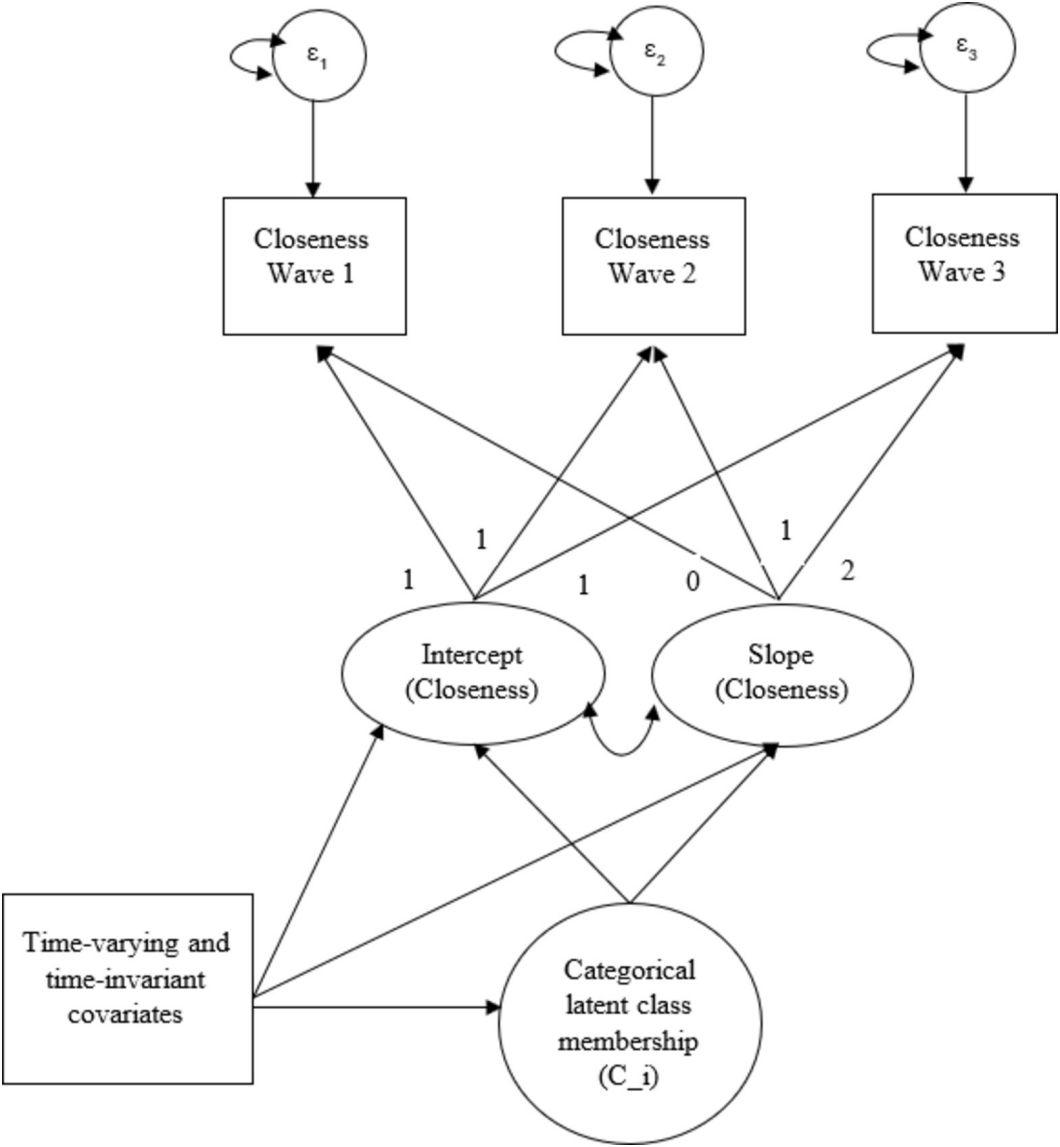
Path diagram for the conditional growth mixture model of mother‐closeness including time‐varying and time‐invariant covariates. The diagram illustrates the association between the included covariate factors (time‐varying and time‐invariant), class membership (latent class group each family belongs to) and levels of mother–child closeness at baseline (intercept) and change over time (slope). Closeness: mother–child closeness.

GMM consists of two parts: (i) class membership and (ii) within‐class trajectories, which are used to estimate parameters for the covariates associated with class membership and within‐class trajectories (Proust‐Lima et al. 2017). For class membership, a multinomial logistic model is used to estimate the probability of membership. Each family belongs to one class, defined by the categorical variable (c_i), which can be determined by time and distinct covariates (Muthén 2004; Proust‐Lima et al. 2017). The second part of GMM specifies the covariates of within‐class trajectories for each class, which are also conditional to time (Proust‐Lima et al. 2017). Unlike traditional growth modelling approaches, using GMM allows for differences in growth parameters and class membership that may be associated with distinct estimates of variance and covariates (Jung and Wickrama 2008). See Figure 1 for the path diagram for this study.

As pre‐registered, we first developed a baseline model to assess changes in mother–child closeness according to time. As displayed in Figure 1, the factor loadings for the latent intercept and slope were fixed to 1, 1, 1 and 0, 1, 2, respectively. Models were fitted to the data by specifying the numbers of classes (Ram and Grimm 2009), and we iteratively tested up to a 4‐class model. Model goodness‐of‐fit was determined by comparing model fit indices and parameter estimates for each model (Ram and Grimm 2009), with lower values on the Akaike information criterion (AIC), Bayesian information criterion (BIC) and sample‐size adjusted BIC (SABIC) indicative of better model fits (Nylund et al. 2007). Models were restricted to linear trajectories as only three waves of data were available.

To examine what factors are associated with class membership and within‐class trajectories respectively, we added the proposed covariates to the models and iteratively tested up to a 4‐class model. However, we experienced model convergence issues due to over‐parameterisation (i.e., there was not enough data to support the complexity of the models), and as a result, we had to deviate from the pre‐registration. To address these problems, we first removed random slopes from the models to consider whether these problems were due to the random effects structure. As a second step, we reduced the complexity of the model by reducing the number of covariates. The research team discussed the relative theoretical importance of each proposed covariate, which led to the removal of two covariates (inter‐parental relationship satisfaction and family economic adversity) from the analysis. Additionally, we used the SDQ total difficulties score rather than measuring child behaviour and emotional problems as separate constructs, as originally pre‐registered. Upon re‐running the models with the inclusion of random slopes, we continued to experience model convergence problems with the 4‐class conditional models. Therefore, we removed the 4‐class model from the conditional GMM analyses and continued to iteratively test up to a 3‐class model. Similar to the baseline models, we compared fit indices and parameter estimates to examine model goodness‐of‐fit. Further information about this process can be found in the Supporting Information.

As a final step, we conducted a sensitivity analysis to consider whether controlling for the child's age at Wave 1 would influence any change over time in mother–child closeness. However, as we did not pre‐register for this, we report the findings for this analysis in Table S1.

For the current sample, missing data ranged from 0% (child age at Wave 1 and Wave 3, diagnosis of autism) to 5.7% (mother–child closeness at Wave 1). Data were considered missing completely at random as determined by Little's Missing Completely At Random (MCAR) test, *χ* ^2^(664) = 696, *p* = 0.19. Compared with families who did not take part in all three waves, the amount of missingness in the current sample did not contribute to any systematic differences in mother–child closeness or any of the covariate factors measured at Wave 1. Further details on these comparisons can be found in Tables S2 and S3.

## 3 Results

### 3.1 Descriptive Statistics

Table 2 displays the means, standard deviations and McDonald's omega coefficients for mother–child closeness and the time‐varying covariates at all three study waves. As shown in Table 2, we found that on average mother–child closeness remained relatively consistent at all three waves.

**TABLE 2:** Mean, standard deviation and omega coefficient for mother–child closeness and time‐varying covariates at all three waves.

Variable	Wave 1			Wave 2			Wave 3		
	M	SD	ω	M	SD	ω	M	SD	ω
Outcome									
Mother–child closeness	26.21	4.95	0.78	26.83	5.20	0.77	26.74	5.43	0.80
Time‐varying covariates									
Child behaviour and emotional problems	20.89	6.08	0.82	21.19	6.32	0.79	20.63	6.32	0.80
Child prosocial behaviour	3.83	2.75	0.82	4.21	2.83	0.83	4.33	2.81	0.84
Maternal psychological distress	8.90	5.49	0.88	8.76	5.26	0.87	9.07	5.71	0.90

### 3.2 Baseline Models

The findings from the baseline models measuring mother–child closeness (with time as the only predictor) suggested that all models fitted to the data without any convergence problems (see Table 3). Therefore, we proceeded to fit the models with the inclusion of the proposed covariates. Tables S4–S7 present the parameter estimates for all four class models.

**TABLE 3:** Fit statistics for baseline growth mixture models of mother‐child closeness with time as the only predictor for all class models.

G	loglik	npm	AIC	BIC	SABIC	%class1	%class2	%class3	%class4
gmm1_2	−2900.24	6	5812.49	5835.69	5816.65	100	—	—	—
gmm2_2	−2880.44	10	5780.88	**5819.54**	5787.82	50.99	49.01	—	—
gmm3_2	−2871.61	14	5771.21	5825.34	5780.93	16.71	20.96	62.32	—
gmm4_2	−2865.91	18	**5767.82**	5837.41	**5780.31**	21.53	39.38	7.65	31.44

*Note:* Values highlighted in bold indicate the class with the best model fit.

Abbreviations: AIC = Akaike information criterion; BIC = Bayesian information criterion; loglik = log‐likelihood; npm = number of parameters; SABIC = sample‐size adjusted Bayesian information criterion.

### 3.3 Mother–Child Closeness and Covariates of Within‐Class Trajectories

Overall, the model fit statistics for the conditional models of mother–child closeness (including the within‐class covariates) demonstrated inconsistent findings. More specifically, no single class model clearly demonstrated best goodness‐of‐fit. As shown in Table 4, we found very little difference between the AIC and SABIC values for the 2‐class model (AIC = 5446.24; SABIC = 5460.12) and the 3‐class model (AIC = 5441.21; SABIC = 5461.34). Both the AIC and SABIC values for the 1‐class model indicated a poorer model fit compared with the other models (AIC = 5475.20; SABIC = 5482.79). However, the BIC value for the 1‐class model (BIC = 5517.69) suggested better model fit compared with the 2‐class (BIC = 5523.57) and 3‐class model (BIC = 5553.34). Sinha et al. (2021) recommend either the BIC or SABIC value as a more reliable indicator of goodness‐of‐model fit, unless the sample size is less than 300 participants or the model is highly complex in which case it is best to draw upon the AIC and BIC.

**TABLE 4:** Fit statistics for the conditional growth mixture models of mother–child closeness and covariates of within‐class trajectories for all class models.

G	loglik	npm	AIC	BIC	SABIC	Entropy	%class1	%class2	%class3
gmm1_2WCP	−2726.58	11	5475.20	**5517.69**	5482.79	1.00	100	—	—
gmm2_2WCP	−2703.20	20	5446.24	5523.57	**5460.12**	0.52	42.78	57.22	—
gmm3_2WCP	−2691.61	29	**5441.21**	5553.34	5461.34	0.44	24.93	30.03	45.04

*Note:* Values highlighted in bold indicate the class with the best model fit.

Abbreviations: AIC = Akaike information criterion; BIC = Bayesian information criterion; loglik = log‐likelihood; npm = number of parameters; SABIC = sample‐size adjusted Bayesian information criterion.

We also examined the entropy values that indicate accuracy of class definition by the model and the proportions of families classified into each class group for all models. We found that the entropy values for the 2‐class (0.52) and 3‐class models (0.44) indicated poor model fit. Typically, an entropy value between 0.80 and 1.00 is considered acceptable (Nylund et al. 2007). These poor model fits, coupled with the inconsistent fit indices, suggest that distinct subgroup trajectories of mother–child closeness were not supported by the data (i.e., the subgroup structure is being forced upon the data rather actually explaining the underlying heterogeneity). Furthermore, although the 2‐class model showed a marginally better entropy value than the 3‐class, the proportion of families classified into Class 1 and Class 2 (Class 1 = 42.78%; Class 2 = 57.22%) was relatively evenly distributed. Therefore, this likely forced dichotomy, coupled with poor model fit, is indicative of the modelling process artificially dividing the sample into two separate groups. As such, we concluded that the 1‐class model best fits the data.

As pre‐registered, we also examined and report the results for the parameter estimates to provide additional information about each class model beyond that presented in the model fit statistics. Tables S8 and S9 present the parameter estimates for the 2‐ and 3‐class models.

Given that the findings suggested there seems to be a single closeness trajectory, we continued to model the conditional models including mother–child closeness and the covariates of the within‐class trajectories and class membership. The purpose of this was to examine whether adding the class membership covariates refined the classes or added further evidence to our suggestion of a single trajectory.

### 3.4 Mother–Child Closeness and Covariates of Within‐Class Trajectories and Class Membership

The results for the conditional model fit statistics, including covariates for within‐class trajectories and class membership, further suggested that distinct subgroup trajectories of mother–child closeness were not supported by the data. As shown in Table 5, the findings for the fit indices indicated some small but noticeable improvements when adding the class membership covariates to the models. For instance, we found minor improvements in the AIC and SABIC values for the 2‐ and 3‐class models compared with the 1‐class model. As with the previous models, the 1‐class model showed a better model fit on the BIC compared with the 2‐ and 3‐class models. Therefore, despite these improvements in fit indices for the 2‐ and 3‐class models, we continued to find that there were inconsistencies in the fit statistics for all class models.

**TABLE 5:** Fit statistics for conditional growth mixture models of closeness with within class predictors and class specific predictors for all class models.

G	loglik	npm	AIC	BIC	SABIC	Entropy	%class1	%class2	%class3
gmm1_2WCPS	−2726.58	11	5475.20	**5517.69**	5482.79	1.00	100	—	—
gmm2_2WCPS	−2691.73	25	5433.45	5530.11	**5450.80**	0.56	45.61	54.39	—
gmm3_2WCPS	−2674.99	39	**5427.98**	5578.78	5455.05	0.68	47.03	49.01	3.97

*Note:* Values highlighted in bold indicate the class with the best model fit.

Abbreviations: AIC = Akaike information criterion; BIC = Bayesian information criterion; loglik = log‐likelihood; npm = number of parameters; SABIC = sample‐size adjusted Bayesian information criterion.

Once more, we examined the entropy value and %class for all models. Interestingly, both the 2‐ and 3‐class models displayed higher entropy values (2‐class: 0.56; 3‐class: 0.68), indicating some improvement but still insufficient for satisfactory model fit. For the 3‐class model, only a very small proportion of families were assigned to class 3 (3.97%), suggesting that the data were not fitted to the model correctly (Ram and Grimm 2009). Therefore, we suggest that these improvements in model fits are instead because the models have more flexibility given the additional parameters introduced, rather than demonstrating better fit to the data. In addition, the proportion of families for the 2‐class model similarly demonstrated equal proportions between each class group (Class 1 = 45.61%; Class 2 = 54.39%), which, in conjunction with the entropy estimates, further suggests that these findings are inconclusive and these families are likely on a continuum which is artificially dichotomised.

The parameter estimates for covariates in the class membership models also confirmed that these models followed a similar pattern to the previous conditional models including within‐class trajectory covariates only (see Tables S10 and S11). In light of these findings, we focussed on the results from the 1‐class model of mother–child closeness in families of children with intellectual disabilities.

As shown in Table 6, we found that the average initial levels of mother–child closeness at Wave 1 were significant (*b =* 22.17, SE = 0.74, *p* = < 0.001). However, there was no significant change in mother–child closeness over time (*b =* 0.10, SE = 0.11, *p* = 0.39). Although no apparent change over time was observed on average, the initial level of closeness in families of children with intellectual disabilities may be important in distinguishing groups.

**TABLE 6:** Summary of parameter estimates for the 1‐class model including covariates of within‐class trajectories.

	1‐class model		
	Coefficient	SE	*p*
Fixed effects			
Intercept	22.17	0.74	< 0.001***
Time	0.10	0.11	0.39
Child behaviour and emotional problems	−0.05	0.03	0.06
Child prosocial behaviour	0.72	0.06	< 0.001***
Child communication skills	0.55	0.08	< 0.001***
Child diagnosis of autism	−1.37	0.40	0.001***
Maternal psychological distress	−0.07	0.03	0.01**
Random effects			
Random intercept variance (*τ*_00_)	7.38	—	—
Random slope variance (*τ*_11_)	0.99	—	—
Random intercept and slope covariance	0.12	—	—
Residual standard error	2.52	0.10	—

** *p =* < 0.01.

*** *p* = < 0.001.

Despite there being no overall change in closeness over time, on examining the covariate factors in the 1‐class model, we found that there were some factors generally associated with mother–child closeness over time. Increases in maternal psychological distress and a diagnosis of autism were associated with reduced levels of mother‐child closeness over time (psychological distress: *b* = −0.07, SE = 0.03, *p* = 0.01; autism diagnosis: *b* = −1.37, SE = 0.40, *p* = 0.001). Additionally, we found that child prosocial behaviour and the child's level of communication skills were generally associated with increased levels of mother‐child closeness over time (child prosocial behaviour: *b =* 0.72, SE = 0.06, *p* < 0.001; child communication skills: *b =* 0.55, SE = 0.08, *p* < 0.001). Child behavioural and emotional problems were not significantly associated with levels of mother–child closeness over time (*b =* −0.05, SE = 0.03, *p* = 0.06).

Finally, the random effects for the 1‐class model indicated a large amount of heterogeneity in mother–child closeness at Wave 1 (*τ*_00_ = 7.38), but there was smaller variation in change in individual trajectories over time (*τ*_11_ = 0.99). This suggests that families of children with intellectual disabilities initially show greater individual differences in closeness, which implies the presence of two groups of families (high versus low levels of mother–child closeness). However, there was little individual variation in change over time, which is consistent with the average effect and stability of the level of mother‐child closeness over time.

## 4 Discussion

The results from this study identified a single trajectory group of mother–child closeness in families of children with intellectual disabilities, with different factors influencing heterogeneity amongst this group. When examining covariates for the 1‐class model, we found that child prosocial behaviour and the child's level of communication skills were generally associated with increased levels of mother–child closeness over time, whereas maternal psychological distress and an autism diagnosis were generally associated with less closeness over time. Child behavioural and emotional problems were not associated with changes in mother–child closeness over time.

The identification of a single trajectory of mother–child closeness in the current study contrasts with previous research examining subgroup trajectories of maternal warmth/closeness in families of non‐disabled children. Both Buckley et al. (2024) and Seiffge‐Krenke et al. (2010) reported three distinct trajectories of mother–child warmth/closeness in families of non‐disabled children when examining data collected at four and seven time points, respectively. One reason for this difference in findings may be because these studies controlled for age at each time point and analysed data across more waves, which is beneficial for identifying distinct trajectory shapes. Moreover, the relationship between the mother and child in this population may be more susceptible to change during childhood as the child gains independence, forms more peer relationships and spends less time within the family home. Additionally, Buckley et al. used group‐based trajectory modelling which assumes trajectory subgroups are homogenous and does not allow for within‐class variation (Nguena Nguefack et al. 2020), which was a key finding in the current study.

Our findings also showed that, although we identified a single trajectory of mother–child closeness, there was no significant average change in closeness over time. However, we found significant heterogeneity in mother‐closeness at baseline and over time, highlighting that individual differences may be more important to consider than average trajectory growth. This finding is consistent with previous research by Fielding‐Gebhardt et al. (2020) and, using the same sample of families as the current study, Taylor et al. (2026). One possible explanation for this finding is that children with intellectual disabilities continue to rely on maternal support into adulthood; thus, changes in mother–child relationship might occur over a longer period of time compared to families of children without intellectual disabilities. Additionally, the samples across these studies were demographically similar (e.g., predominantly Caucasian, married, educated to degree level), which may have influenced the similarities in findings. Alternatively, the measures used may not be sensitive enough to detect average rates of change over time in these families. However, as the findings from this study indicated that there could be two groups of families (high versus low closeness), future research should consider using methods not based on the average effect but conditionally across the whole distribution to determine different effects associated at different levels of mother–child closeness (Yu et al. 2003).

The inclusion of covariate factors helped account for individual differences in mother–child closeness over time. Consistent with previous research (e.g., Teague et al. 2020; Totsika et al. 2020), maternal psychological distress and a diagnosis of autism were associated with reduced levels of closeness over time. Similar to Taylor et al. (2026), the child's level of communication skills and child prosocial behaviour were associated with increases in mother–child closeness over time. One reason for this latter finding may be that greater child prosocial behaviour and child communication skills facilitate more positive interactions and stronger emotional bonds between the parent and child. However, unlike Fielding‐Gebhardt et al. (2020), child behaviour and emotional problems were not associated with levels of closeness over time. This finding may be due to differences in analytical models and other covariates controlled for, which in the current study were significantly associated with mother–child closeness over time.

Despite the current study being a novel attempt at understanding whether there are distinct trajectories of mother–child closeness in families of children with intellectual disabilities, there were a number of limitations to this study. First, data were only available at three waves, which limited our ability to model for non‐linear trajectories that might have offered additional insights into how mother–child closeness changes over time. Second, slope factor loadings in the models were fixed to 0, 1, 2, which assumes equal spacing between time intervals. Like many longitudinal studies, recruitment periods in the original study varied at each wave, meaning that the time intervals were not always equal across all participants. Although the current time coding was used as the best approximation, it is possible that there may be some imprecisions with this time coding, which reflects the practical complexity of recruiting and retaining families in longitudinal studies. However, it is unlikely this coding substantially influenced the current findings. Third, the sample was predominantly White British (88%), which limits the generalisability of these findings to more diverse populations. Fourth, this study relied solely on mother‐reported data; including the perspectives of fathers or children themselves may yield a better understanding of the parent–child relationship in these families. Finally, this study included a broad age range for children at Wave 1 (between 4 years and 15 years and 11 months). Although our sensitivity analysis suggested that age did not influence levels of closeness in this sample, future research should still consider analysing data from birth population cohort studies. In light of the findings from this study suggesting that there may be two groups of families, further research addressing these limitations is needed when examining for subgroup trajectories of mother–child closeness in families of children with intellectual disabilities.

The findings also highlight the importance of providing early intervention support to mothers who may feel less close to their child to enhance this relationship over time and, in turn, reduce the risk of poorer child outcomes. Targeted support may be particularly important for families with children with intellectual disabilities with lower communication skills, fewer displays of prosocial behaviour or who may also be autistic. Moreover, this support may be important for mothers who experience increased mental health difficulties themselves.

## Funding

E.L.T. is funded by the Economic and Social Research Council Midlands Graduate School Doctoral Training Partnership as part of a funded PhD studentship (ES/P00711/1). The 1000 Families Study was funded by Cerebra UK, the Economic and Social Research Council Warwick Doctoral Training Centre and the University of Warwick.

## Ethics Statement

Ethics approval for the present study was received by the Humanities and Social Sciences Research Ethics Committee at the University of Warwick (Reference Number: HSSREC.222/23‐24).

## Conflicts of Interest

The authors declare no conflicts of interest.

## Supporting information


**Table S1:** Summary of parameter estimates for the 1‐class model including covariates of within‐class trajectories—sensitivity analysis for child's age at Wave 1.
**Table S2:** Comparative summary of missing data for families who did and did not take part in all three waves.
**Table S3:** Comparative summary of baseline descriptive statistics for families who did and did not take part in all three waves.
**Table S4:** Summary of parameter estimates for the unconditional 1‐class model according to time.
**Table S5:** Summary of parameter estimates for the unconditional 2‐class model according to time.
**Table S6:** Summary of parameter estimates for the unconditional 3‐class model according to time.
**Table S7:** Summary of parameter estimates for the unconditional 4‐class model according to time.
**Table S8:** Summary of parameter estimates for the 2‐class model including covariates of within‐class trajectories.
**Table S9:** Summary of parameter estimates for the 3‐class model including covariates of within‐class trajectories.
**Table S10:** Summary of parameter estimates for the 2‐class model including covariates of within‐class trajectories and class membership.
**Table S11:** Summary of parameter estimates for the 3‐class model including covariates of within‐class trajectories and class membership.

## Data Availability

Due to ethic approval requirements, data are not available for sharing. Researchers who are interested in collaborating should contact Professor Richard Hastings directly.
