# Supplementary material for: Mother–Child Closeness Trajectories in Families of Children With Intellectual Disabilities From a UK Cohort Study
Source: J Intellect Disabil Res. 2026 May 27;70(8):835–44. doi: 10.1111/jir.70122 (PMC13327159; doi:10.1111/jir.70122)
Supplement: Supplementary file 1 — Table S1: Summary of parameter estimates for the 1‐class model including covariates of within‐class trajectories—sensitivity analysis for child's age at Wave 1. Table S2: Comparative summary of missing data for families who did and did not take part in all three waves. Table S3: Comparative summary of baseline descriptive statistics for families who did and did not take part in all three waves. Table S4: Summary of parameter estimates for the unconditional 1‐class model according to time. Table S5: Summary of parameter estimates for the unconditional 2‐class model according to time. Table S6: Summary of parameter estimates for the unconditional 3‐class model according to time. Table S7: Summary of parameter estimates for the unconditional 4‐class model according to time. Table S8: Summary of parameter estimates for the 2‐class model including covariates of within‐class trajectories. Table S9: Summary of parameter estimates for the 3‐class model including covariates of within‐class trajectories. Table S10: Summary of parameter estimates for the 2‐class model including covariates of within‐class trajectories and class membership. Table S11: Summary of parameter estimates for the 3‐class model including covariates of within‐class trajectories and class membership. [file JIR-70-835-s001.docx]

**Supplementary Material**

**Summary Description of Modelling Convergence Issue**

The following paragraph provides an overview of the model convergence issues we experienced when running the original pre-registered statistical analysis plan, as detailed in the main manuscript. In summary, we pre-registered that we would model up to a 4-class model for both the unconditional and conditional models. For the conditional models, we planned to include: child externalising behaviour problems, child internalising behaviour problems, child prosocial behaviour, a diagnosis of autism, the child’s level of communication skills, family economic adversity, inter-parental relationship satisfaction, and maternal psychological distress as predictors of within-class trajectories and class membership. As we did not experience convergence issues for the unconditional models, we will focus on the conditional models. To note, the term ‘conditional models’ is used to describe both the within-class and class membership models unless specified otherwise.

Upon running the conditional models, we experienced model convergence issues with the 2-, 3-, and 4- class models. With the 3-and 4-class models, we received error messages detailing that the maximum number of iterations had been exceeded. Therefore, indicating that the model was unable to successfully meet the convergence criteria. As such, we were unable to acquire an output for these models. In contrast, despite appearing to have converged due to the lack of error message, the summary output for the 2-class model contained missing information which is also indicative of model convergence issues.

As a first step, we followed our pre-registration and removed the random slopes from the conditional models to remove a layer of complexity. By removing the random slopes from the model, it allowed us to reduce the number of parameters the model was having to account for. However, upon re-running the models with the removal of the random slopes, we continued to receive the same error message for the 3- and 4-class models. Therefore, we decided to deviate from the pre-registration to remove an additional layer of complexity from these models.

As a next step, we decided to reduce the number of covariates included the models. As a research team, we discussed the theoretical importance of each covariate in detail before ranking and grouping them based on their importance. The purpose of this was to systematically add “blocks” of covariates into the models to consider the threshold of model complexity and ensuring that we were including covariates that were most appropriate. During our discussion, we also decided to create a total child behaviour problems variable rather than use separate child externalising and internalising behaviour problems to remove this additional parameter. These blocks were: 1) child prosocial behaviour, maternal psychological distress; child’s level of communication skills; diagnosis of autism; child behaviour problems; 2) family economic adversity; inter-parental relationship satisfaction. Upon re-running the conditional models (still without the inclusion of random slopes), we found that the 2-and 3-class models converged with the inclusion of block one covariates only. However, we continued to experience convergence issues with the 4-class model.

Since we were still experiencing model convergence issues for the 4-class conditional models, we reviewed the fit indices again for the unconditional models. We found that there was very little difference between the AIC and BIC values for 3-and 4-class models. In addition, the 4-class model contained a very small %class (7.65%). As this study is a data driven approach, we concluded that the 4-class model was unlikely to best describe the data and therefore decided to remove this class model from the analysis.

As a final step, we re-ran the conditional models (excluding the 4-class model) with the inclusion of the random slopes. All models successfully converged with the final results presented in the main manuscript.

**Sensitivity Analysis Controlling for Children’s Age at Wave 1**

We conducted a sensitivity analysis to examine whether controlling for the child’s age at Wave 1 would contribute to any change in the trajectory growth of mother-child closeness. Based on the findings from the main analysis, which indicated that the 1-class model provided the best model fit, we added the child’s age at Wave 1 into this model as a time-invariant covariate. At Wave 1, the age of the children ranged between 4 years and 15 years 11 months (M=7.33 years).

As shown in Table S1, we found that controlling for the child’s age at Wave 1 did not change the average rates of growth in mother-child closeness over time; mother-child closeness remained stable over time. The child’s age was not significantly associated with mother-child closeness over time. Moreover, the estimates, standard errors and p-values for the pre-existing mother- and child-related covariates showed no substantial changes and therefore suggests that age did not substantially contribute to the model in explaining the trajectory of mother-child closeness. In addition, the estimates for the random effects showed little to no change compared to the model in the main analysis, indicating that controlling for children’s age did not account for heterogeneity amongst this group of families.

**Table S1**

*Summary of Parameter Estimates for the 1-Class Model including Covariates of Within-Class Trajectories – Sensitivity Analysis for Child’s Age at Wave 1*

|  | **1-Class Model** | | |  |
| --- | --- | --- | --- | --- |
| **Fixed Effects** | **Coefficient** | **SE** | ***p*** | |
| Intercept | 22.96 | 0.95 | <.001*** | |
| Time | 0.09 | 0.11 | .44 | |
| Child Behaviour and Emotional Problems | -0.04 | 0.03 | .14 | |
| Child Prosocial Behaviour | 0.73 | 0.06 | <.001*** | |
| Child Communication skills | 0.56 | 0.09 | <.001*** | |
| Child Diagnosis of Autism | -1.43 | 0.41 | <.001*** | |
| Maternal Psychological Distress | -0.07 | 0.03 | .01** | |
| Child Age (Wave 1) | -0.14 | 0.09 | .13 | |
| **Random Effects** |  |  |  | |
| Random intercept variance (τ_00_) | 7.45 | - | **-** | |
| Random slope variance (τ_11_) | 0.98 | - | **-** | |
| Random intercept and slope covariance | 0.12 | - | **-** | |
| **Residual Standard Error** | 2.52 | 0.10 | **-** | |

*Note.* ****p*=<.001; ***p*=<.01

**Comparison Between the Current Sample and Families Who Did Not Take Part at All Three Waves**

We compared the extent of missing data for each variable in both the current sample (i.e., families who participated in all three waves) and in a sample of families who did not take part in all three waves. The inclusion criteria for the current sample were: 1) the main respondent must identify as a maternal caregiver at any of the three waves, and 2) their child with intellectual disabilities must be under the age of 16 years at Wave 3, as the survey administered at this Wave for children over the age of 16 years did not include a measure of child behaviour and emotional problems. To include families who did not take part in all three Waves, we first filtered for main respondents who identified as a maternal caregiver at Wave 1 and Wave 2. However, because some of these data were missing at Wave 2, we retained families where identification as a maternal caregiver were available at Wave 1 but not Wave 2. Furthermore, because not all families took part in Wave 3, our initial criteria risked excluding children who would have been under the age of 16 years at Wave 1. Therefore, to mitigate excluding these families, we took a pragmatic approach and excluded children who were over the age of 11.35 years at Wave 1. This age represents the maximum Wave 1 age that would make a child eligible for the under 16 survey at Wave 3, based on the descriptive statistics for age at Wave 1 for the sample used in the current study. A total of 731 families were included in this new dataset.

As shown in Table S2, the overall amount of missingness in this new sample was 23.8%, compared to 1.8% in the original dataset used for the current study. However, across individual variables, this missingness ranged between 0% (child age at Wave 1) and 55.13% (child’s level of communication skills at Wave 3). Table S3 provides a comparison of descriptive statistics between these samples in key variables at Wave 1.

**Table S2**

*Comparative Summary of Missing Data for Families Who Did and Did Not Take Part in all Three Waves*

| **Variable** | **Missingness (%) for families who did not take part in all three Waves** | **Missingness (%) for families who did take part in all three Waves** |
| --- | --- | --- |
| **Demographics** |  |  |
| Child Sex – Wave 1 | 2 (0.2%) | 2 (0.6%) |
| Ethnicity – Wave 1 | 6 (0.8%) | 6 (1.7%) |
| Child Age – Wave 1 | 0 (0%) | 0 (0%) |
| Child Age – Wave 2 | 236 (32.3%) | 2 (0.6%) |
| Child Age – Wave 3 | 326 (44.6%) | 0 (0%) |
| Caregiver’s Level of Education – Wave 1 | 35 (4.8%) | 12 (3.4%) |
| Caregiver’s Job – Wave 1 | 1 (0.1%) | 1 (0.3%) |
| Caregiver’s Marital Status – Wave 1 | 14 (1.92%) | 16 (4.5%) |
| **Outcome and Covariates** |  |  |
| Mother-Child Closeness – Wave 1 | 29 (3.97%) | 20 (5.7%) |
| Mother-Child Closeness – Wave 2 | 241 (32.97%) | 6 (1.7%) |
| Mother-Child Closeness – Wave 3 | 400 (54.72%) | 2 (0.6%) |
| Maternal Psychological Distress – Wave 1 | 10 (1.37%) | 15 (4.2%) |
| Maternal Psychological Distress – Wave 2 | 237 (32.42%) | 3 (0.8%) |
| Maternal Psychological Distress – Wave 3 | 328 (44.87%) | 2 (0.6%) |
| Child Prosocial Behaviour – Wave 1 | 21 (2.87%) | 20 (5.7%) |
| Child Prosocial Behaviour – Wave 2 | 238 (32.56%) | 6 (1.7%) |
| Child Prosocial Behaviour – Wave 3 | 399 (54.58%) | 1 (0.3%) |
| Child Behaviour and Emotional Problems – Wave 1 | 21 (2.87%) | 20 (5.7%) |
| Child Behaviour and Emotional Problems – Wave 2 | 239 (32.69%) | 7 (2.0%) |
| Child Behaviour and Emotional Problems – Wave 3 | 400 (54.72%) | 2 (0.6%) |
| Child Diagnosis of Autism | 102 (13.95%) | 0 (0%) |
| Child’s Level of Communication – Wave 2 | 245 (33.52%) | 9 (2.5%) |
| Child’s Level of Communication – Wave 3 | 403 (55.13%) | 5 (1.4%) |

**Table S3**

*Comparative Summary of Baseline Descriptive Statistics for Families Who Did and Did Not Take Part in all Three Waves*

| **Variable** | **Families who did take part in all three Waves** | **Families who did not take part in all three Waves** |
| --- | --- | --- |
|  | **M (SD)** | **M (SD)** |
| Mother-child closeness | 26.21 (4.95) | 25.83 (5.19) |
| Child behaviour problems | 20.89 (6.08) | 21.09 (6.48) |
| Child prosocial behaviour | 3.83 (2.75) | 3.83 (2.80) |
| Maternal psychological distress | 8.9 (5.49) | 9.28 (5.54) |
|  | **N** | **N** |
| Autism | 236 | 459 |

**Table S4**

*Summary of Parameter Estimates for the Unconditional 1-Class Model According to Time*

|  | **1-Class Model** | | |
| --- | --- | --- | --- |
| **Fixed Effects** | **Coefficient** | **SE** | ***p*** |
| Intercept | 26.29 | 0.26 | <.001******* |
| Time | 0.28 | 0.12 | .02* |
| **Random Effects** |  |  |  |
| Random intercept variance (τ_00_) | 18.02 | - | **-** |
| Random slope variance (τ_11_) | 1.44 | - | **-** |
| Random intercept and slope covariance | -0.16 | - | **-** |
| **Residual Standard Error** | 2.61 | 0.10 | **-** |

*Note.* ****p* = <.001, **p=* <.05

**Table S5**

|  | **1-Class Model (n=180)** | | | | **2-Class Model (n=173)** | | |
| --- | --- | --- | --- | --- | --- | --- | --- |
| **Mean Probability of Class Membership** |  | .78 |  |  | | .88 |  |
| **Fixed Effects** | **Coefficient** | **SE** | ***p*** | **Coefficient** | | **SE** | ***p*** |
| **Class-Membership Model*** |  |  |  |  | |  |  |
| Intercept | -0.18 | 0.35 | .62 | - | | - | - |
| **Longitudinal Model** |  |  |  |  | |  |  |
| Intercept | 29.65 | 0.50 | <.001*** | 23.47 | | 0.76 | <.001*** |
| Time | 0.24 | 0.24 | .31 | 0.31 | | 0.23 | .18 |
| **Random Effects** |  |  |  |  | |  |  |
| Random intercept variance (τ_00_) | 3.28 | - | **-** | 14.09 | | - | - |
| Random slope variance (τ_11_) | 0.62 | - | **-** | 2.68 | | - | - |
| Random intercept and slope covariance | -0.14 | - | **-** | -0.60 | | - | - |
| Proportional coefficient | 0.48 | 0.09 | **-** | - | | - | - |
| **Residual Standard Error** | 2.55 | 0.09 | **-** | 2.55 | | 0.09 | - |

*Summary of Parameter Estimates for the Unconditional 2-Class Model According to Time*

*Note.* *Class 2 is the reference group; ****p* = <.001

**Table S6**

*Summary of Parameter Estimates for the Unconditional 3-Class Model According to Time*

|  | **1-Class Model (n=59)** | | | **2-Class Model (n=74)** | | | **3-Class Model (n=220)** | | |
| --- | --- | --- | --- | --- | --- | --- | --- | --- | --- |
| **Mean Probability of Class Membership** |  | .84 |  |  | .79 |  |  | .87 |  |
| **Fixed Effects** | **Coefficient** | **SE** | ***p*** | **Coefficient** | **SE** | ***p*** | **Coefficient** | **SE** | ***p*** |
| **Class-Membership Model*** |  |  |  |  |  |  |  |  |  |
| Intercept | -1.22 | 0.39 | .002** | -1.08 | 0.30 | <.001*** | - | - | - |
| **Longitudinal Model** |  |  |  |  |  |  |  |  |  |
| Intercept | 20.70 | 1.07 | <.001*** | 31.46 | 0.42 | <.001*** | 26.18 | 0.47 | <.001*** |
| Time | -0.96 | 0.53 | .07 | 0.422 | 0.26 | .11 | 0.60 | 0.19 | .002** |
| **Random Effects** |  |  |  |  |  |  |  |  |  |
| Random intercept variance (τ_00_) | 12.69 | - | **-** | 0.00 | - | - | 8.36 | - | - |
| Random slope variance (τ_11_) | 2.70 | - | **-** | 0.00 | - | - | 1.78 | - | - |
| Random intercept and slope covariance | -3.42 | - | **-** | -0.00 | - | - | -2.25 | - | - |
| Proportional coefficient | 1.23 | 0.30 | **-** | 0.01 | 0.28 | - | - | - | - |
| **Residual Standard Error** | 2.52 | 0.09 | **-** | 2.52 | 0.09 | - | 2.52 | 0.09 | - |

*Note*. *Class 3 is the reference group; *** *p* =<.001, ***p* =<.01

| **Table S7**  *Summary of Parameter Estimates for the Unconditional 4-Class Model According to Time* | **1-Class Model (n=76)** | | | | | **2-Class Model (n=27)** | | | | **3-Class Model (n=139)** | | | | | | **4-Class Model (n=111)** | | |  |
| --- | --- | --- | --- | --- | --- | --- | --- | --- | --- | --- | --- | --- | --- | --- | --- | --- | --- | --- | --- |
| **Mean Probability of Class Membership** |  | .88 | |  | | .91 | |  |  | |  | |  | .81 | | .85 | | |  |
| **Fixed Effects** | **Coefficient** | | **SE** | ***p*** | **Coefficient** | | **SE** | ***p*** | | | | **Coefficient** | | **SE** | ***p*** | **Coefficient** | **SE** | ***p*** | |
| **Class-Membership Model*** |  | |  |  |  | |  |  | | | |  | |  |  |  |  |  | |
| Intercept | -0.33 | | 0.30 | .28 | -1.32 | | 0.36 | <.001*** | | | | 0.118 | | 0.23 | .42 | - | - | - | |
| **Longitudinal Model** |  | |  |  |  | |  |  | | | |  | |  |  |  |  |  | |
| Intercept | 31.59 | | 0.42 | <.001*** | 17.42 | | 0.62 | <.001*** | | | | 27.91 | | 0.44 | <.001*** | 22.87 | 0.47 | <.001*** | |
| Time | 0.38 | | 0.24 | .11 | -0.39 | | 0.63 | .54 | | | | 0.14 | | 0.24 | .55 | 0.55 | 0.29 | .05* | |
| **Random Effects** |  | |  |  |  | |  |  | | | |  | |  |  |  |  |  | |
| Random intercept variance (τ_00_) | 0.00 | | - | **-** | 1.46 | | - | - | | | | 0.62 | | - | - | 2.30 | - | - | |
| Random slope variance (τ_11_) | 0.00 | | - | **-** | 2.53 | | - | - | | | | 1.08 | | - | - | 3.99 | - | - | |
| Random intercept and slope covariance | -0.00 | | - | **-** | -1.19 | | - | - | | | | -0.51 | | - | - | -1.87 | - | - | |
| Proportional coefficient | 0.00 | | 0.06 | **-** | 0.80 | | 0.31 | - | | | | 0.52 | | 0.16 | - | - | - | - | |
| **Residual Standard Error** | 2.52 | | 0.09 | **-** | 2.52 | | 0.09 | - | | | | 2.52 | | 0.09 | - | 2.52 | 0.94 | - | |

*Note.* *****Class 4 is the reference group; *** *p*=<.001; **p*=<.05**Table S8**

*Summary of Parameter Estimates for the 2-Class Model including Covariates of Within-Class Trajectories*

|  | **1-Class Model (n=151)** | | | **2-Class Model (n=202)** | | |
| --- | --- | --- | --- | --- | --- | --- |
| **Mean Probability of Class Membership** |  | .79 |  |  | .89 |  |
| **Fixed Effects** | **Coefficient** | **SE** | ***p*** | **Coefficient** | **SE** | ***p*** |
| **Class-Membership Model*** |  |  |  |  |  |  |
| Intercept | -0.40 | 0.21 | .07 | - | - | - |
| **Longitudinal Model** |  |  |  |  |  |  |
| Intercept | 25.60 | 0.96 | <.001*** | 20.85 | 0.90 | <.001*** |
| Time | 0.16 | 0.19 | .40 | 0.07 | 0.18 | .67 |
| Child Behaviour and Emotional Problems | -0.03 | 0.04 | .37 | -0.07 | 0.03 | .04* |
| Child Prosocial Behaviour | 0.57 | 0.08 | <.001*** | 0.79 | 0.08 | <.001*** |
| Child Communication Skills | 0.34 | 0.10 | .001*** | 0.60 | 0.11 | <.001*** |
| Child Diagnosis of Autism | -0.55 | 0.45 | .22 | -1.79 | 0.51 | <.001*** |
| Maternal Psychological Distress | -0.01 | 0.03 | .71 | -0.11 | 0.04 | .002** |
| **Random Effects** |  |  |  |  |  |  |
| Random intercept variance (τ_00_) | 0.68 | - | **-** | 4.05 | - | - |
| Random slope variance (τ_11_) | 0.35 | - | **-** | 2.10 | - | - |
| Random Intercept and Slope Covariance | -0.07 | - | **-** | -0.44 | - | - |
| Proportional coefficient | 0.41 | 0.12 | **-** | - | - | - |
| **Residual Standard Error** | 2.44 | 0.09 | **-** | 2.44 | 0.09 | - |

*Note.* *Class 2 is the reference group; ****p=<*.001; ***p*=<.01; **p*=<.05

**Table S9**

*Summary of Parameter Estimates for the 3-Class Model including Covariates of Within-Class Trajectories*

|  | **1-Class Model (n=88)** | | | **2-Class Model (n=106)** | | | **3-Class Model (n=159)** | | |
| --- | --- | --- | --- | --- | --- | --- | --- | --- | --- |
| **Mean Probability of Class Membership** |  | .75 |  |  | .63 |  |  | .76 |  |
| **Fixed Effects** | **Coefficient** | **SE** | ***p*** | **Coefficient** | **SE** | ***p*** | **Coefficient** | **SE** | ***p*** |
| **Class-Membership Model*** |  |  |  |  |  |  |  |  |  |
| Intercept | -0.12 | 0.35 | .74 | -0.33 | 0.25 | .19 | - | - | - |
| **Longitudinal Model** |  |  |  |  |  |  |  |  |  |
| Intercept | 21.03 | 1.75 | <.001*** | 21.63 | 1.14 | <.001*** | 24.73 | 0.94 | <.001*** |
| Time | -0.17 | 0.33 | .61 | 0.27 | 0.30 | .38 | 0.24 | 0.19 | .21 |
| Child Behaviour and Emotional Problems | -0.08 | 0.07 | .22 | -0.08 | 0.05 | .06 | -0.01 | 0.03 | .76 |
| Child Prosocial Behaviour | 0.63 | 0.15 | <.001*** | 0.89 | 0.11 | <.001*** | 0.65 | 0.08 | <.001*** |
| Child Communication Skills | 0.68 | 0.21 | .001*** | 0.59 | 0.11 | <.001*** | 0.34 | 0.09 | <.001*** |
| Child Diagnosis of Autism | -2.92 | 1.00 | .003** | -1.04 | 0.56 | .06 | -0.09 | 0.47 | .86 |
| Maternal Psychological Distress | 0.01 | 0.07 | .83 | -0.24 | 0.05 | <.001*** | -0.05 | 0.03 | .16 |
| **Random Effects** |  |  |  |  |  |  |  |  |  |
| Intercept variance (τ_00_) | 8.33 | - | **-** | 0.00 | - | - | 0.34 | - | - |
| Slope variance (τ_11_) | 3.82 | - | **-** | 0.00 | - | - | 0.15 | - | - |
| Random intercept and slope covariance | -1.04 | - | **-** | -0.00 | - | - | -0.04 | - | - |
| Proportional coefficient | 4.97 | 3.14 | **-** | 0.00 | 0.15 | - | - | - | - |
| **Residual Standard Error** | 2.41 | 0.08 | **-** | 2.41 | 0.08 | - | 2.41 | 0.08 | - |

*Note.* *Class 3 is the reference group; ****p*=<.001; ***p*=<.01

**Table S10**

*Summary of Parameter Estimates for the 2-Class Model including Covariates of Within-Class Trajectories and Class Membership*

|  | **1-Class Model (n=161)** | | | | **2-Class Model (n=192)** | | |  |
| --- | --- | --- | --- | --- | --- | --- | --- | --- |
| **Mean Probability of Class Membership** |  | .82 |  |  | | .90 |  | |
| **Fixed Effects** | **Coefficient** | **SE** | ***p*** | **Coefficient** | | **SE** | ***p*** | |
| **Class-Membership Model*** |  |  |  |  | |  |  | |
| Intercept | -1.28 | 0.90 | .16 | - | | - | - | |
| Child Behaviour and Emotional Problems | -0.07 | 0.04 | .07 | - | | - | - | |
| Child Prosocial Behaviour | 0.23 | 0.08 | .005** | - | | - | - | |
| Child Communication Skills | 0.09 | 0.11 | .39 | - | | - | - | |
| Child Diagnosis of Autism | -0.02 | 0.51 | .96 | - | | - | - | |
| Maternal Psychological Distress | 0.09 | 0.04 | .01** | - | | - | - | |
| **Longitudinal Model** |  |  |  |  | |  |  | |
| Intercept | 26.97 | 1.18 | <.001*** | 21.39 | | 0.19 | .73 | |
| Time | 0.06 | 0.19 | .73 | 0.18 | | 0.19 | .35 | |
| Child Behaviour and Emotional Problems | -0.03 | 0.04 | .36 | -0.05 | | 0.04 | .18 | |
| Child Prosocial Behaviour | 0.48 | 0.08 | <.001*** | 0.76 | | 0.09 | <.001*** | |
| Child Communication Skills | 0.25 | 0.11 | .03* | 0.48 | | 0.13 | <.001*** | |
| Child Diagnosis of Autism | -0.49 | 0.48 | .31 | -1.50 | | 0.64 | .02* | |
| Maternal Psychological Distress | -0.04 | 0.04 | .20 | -0.14 | | 0.04 | <.001*** | |
| **Random Effects** |  |  |  |  | |  |  | |
| Random intercept variance (τ_00_) | 0.82 | - | **-** | 4.67 | | - | - | |
| Random slope variance (τ_11_) | 0.41 | - | **-** | 2.34 | | - | - | |
| Random intercept and slope covariance | -0.08 | - | **-** | -0.43 | | - | - | |
| Proportional coefficient | 0.42 | 0.10 | **-** | - | | - | - | |
| **Residual Standard Error** | 2.40 | 0.09 | **-** | 2.40 | | 0.09 | - | |

*Note.* *Class 2 is the reference group; ****p*=<.001; ***p*=<.01; **p*=<.05

**Table S11**

*Summary of Parameter Estimates for the 3-Class Model including Covariates of Within-Class Trajectories and Class Membership*

|  | **1-Class Model (n=166)** | | | **2-Class Model (n=173)** | | | **3-Class Model (n=14)** | | |
| --- | --- | --- | --- | --- | --- | --- | --- | --- | --- |
| **Mean Probability of Class Membership** |  | .82 |  |  | .89 |  |  | .90 |  |
| **Fixed Effects** | **Coefficient** | **SE** | ***p*** | **Coefficient** | **SE** | ***p*** | **Coefficient** | **SE** | ***p*** |
| **Class-Membership Model*** |  |  |  |  |  |  |  |  |  |
| Intercept | -2.35 | 1.81 | .19 | -2.56 | 1.95 | .19 | - | - | - |
| Child Behaviour and Emotional Problems | 0.27 | 0.11 | .02* | 0.36 | 0.12 | .002** | - | - | - |
| Child Prosocial Behaviour | 0.32 | 0.17 | .05* | 0.11 | 0.18 | .54 | - | - | - |
| Child Communication Skills | 0.01 | 0.11 | .91 | 0.00 | 0.06 | 1.00 | - | - | - |
| Child Diagnosis of Autism | 0.44 | 1.15 | .70 | 1.11 | 1.35 | .41 | - | - | - |
| Maternal Psychological Distress | -0.15 | 0.11 | .15 | -0.28 | 0.12 | .01** | - | - | - |
| **Longitudinal Model** |  |  |  |  |  |  |  |  |  |
| Intercept | 25.52 | 1.14 | <.001*** | 21.72 | 1.31 | <.001*** | 12.93 | 4.15 | .002** |
| Time | 0.81 | 0.18 | .66 | 0.45 | 0.20 | .02* | -2.73 | 0.89 | .002** |
| Child Behaviour and Emotional Problems | -0.02 | 0.04 | .64 | -0.09 | 0.04 | .02* | 0.26 | 0.14 | .06 |
| Child Prosocial Behaviour | 0.55 | 0.08 | <.001*** | 0.72 | 0.10 | <.001*** | 0.97 | 0.30 | .001*** |
| Child Communication Skills | 0.32 | 0.11 | .01** | 0.55 | 0.14 | <.001*** | 1.23 | 0.50 | .01** |
| Child Diagnosis of Autism | 0.22 | 0.62 | .72 | -1.16 | 0.86 | .18 | -6.51 | 2.85 | .02* |
| Maternal Psychological Distress | -0.08 | 0.04 | .04* | -0.15 | 0.04 | <.001*** | 0.16 | 0.16 | .31 |
| **Random Effects** |  |  |  |  |  |  |  |  |  |
| Intercept variance (τ_00_) | 0.61 | - | **-** | 3.51 | - | - | 8.57 | - | - |
| Slope variance (τ_11_) | 0.22 | - | **-** | 1.26 | - | - | 3.07 | - | - |
| Random intercept and slope covariance | 0.05 | - | **-** | 0.29 | - | - | 0.70 | - | - |
| Proportional coefficient | 0.27 | 0.10 | **-** | 0.64 | 0.14 | - | - | - | - |
| **Residual Standard Error** | 2.40 | 0.09 | **-** | 2.40 | 0.09 | - | 2.40 | 0.09 | - |

*Note.* *Class 3 is the reference group; ****p*=<.001; ***p*=<.01; **p*=<.05
